# High breastfeeding continuation at two months after birth in women receiving home-based postnatal midwifery care and factors associated with breastfeeding cessation: A prospective cohort study

**DOI:** 10.18332/ejm/219215

**Published:** 2026-04-30

**Authors:** Eva Åsman, Christine Rubertsson, Elisabeth Mangrio, Cecilia Häggsgård

**Affiliations:** 1 Department of Health Sciences, Faculty of Medicine, Lund University, Lund, Sweden; 2 Department of Obstetrics and Gynecology, Skåne University Hospital, Malmö, Sweden; 3 Department of Care Science, Faculty of Health and Society, Malmö University, Malmö, Sweden

**Keywords:** breastfeeding cessation, breastfeeding support, exclusive breastfeeding, home-based midwifery care, postnatal care

## Abstract

**INTRODUCTION:**

Exclusive breastfeeding (EB) is globally recommended due to health benefits for women and their infants. Home-based postnatal midwifery care (HBPMC) is a model of care where midwives give in-home care during the first postnatal week. The study objectives were to investigate breastfeeding outcomes at two months after birth, in women and newborns receiving HBPMC, and to identify risk factors and describe women’s reported reasons associated with EB cessation.

**METHODS:**

A prospective cohort study including 219 women in Sweden, conducted between November 2023 and March 2024. Background and breastfeeding variables were collected from medical records. Telephone interviews two months after birth contained open-ended questions about breastfeeding duration and reasons for EB cessation. Multivariable logistic regression was used to calculate adjusted odds ratios (AORs) with 95% confidence intervals (CIs).

**RESULTS:**

In total, 211 women (96%) completed the follow-up. The prevalence of exclusive breastfeeding was 86% at discharge from HBPMC, and 84% at two months after birth. Factors associated with EB cessation included early formula supplementation (AOR=5.89; 95% CI: 2.31–15.02), a pre-pregnancy body mass index ≥ 30 kg/m^2^ (AOR=3.55; 95% CI: 1.27–9.94), and being born in another country (AOR=2.39; 95% CI: 1.01–5.67). The most frequently reported reasons for EB cessation were perceived breastfeeding barriers and insufficient milk supply.

**CONCLUSIONS:**

A high continuation of exclusive breastfeeding was reported at two months after receiving HBPMC, suggesting that in-home breastfeeding support during the first week after birth may play an important role. Future interventions with in-home breastfeeding support targeting women with identified risk factors may further improve breastfeeding duration.

## INTRODUCTION

The World Health Organization (WHO) recommends that all newborns initiate breastfeeding within the first hour of life, to breastfeed exclusively for six months, and thereafter breastfeed with complementary food up to two years and beyond^1^. There is extensive evidence for the health benefits of breastfeeding for mother and child in high- as well as low- and middle-income countries^2,3^. Exclusive breastfeeding (EB), as defined by the WHO, refers to infants receiving only breastmilk with no additional foods or liquids except for vitamins, minerals, or oral medications^4^. Several factors have been identified as contributing to the cessation of EB before six months. These factors are often multi-factorial and encompass sociodemographic characteristics, insufficient partner support, breastfeeding-related difficulties such as suboptimal positioning and ineffective latch, perceived insufficient milk supply, as well as the influence of commercial promotion of infant formula^5^. Introduction of formula only when medical indications occur has been suggested as a critical factor for breastfeeding success^6^. Maternal origin or lack of culturally sensitive support has also been suggested to have an impact on breastfeeding duration^7^.

Data from the Swedish National Board of Health and Welfare in 2022 show an EB rate of 72% at one week after birth, with a significant decrease to 61% at two months, 50% at four months, and 12% at six months after birth^8^. According to a Swedish study, prenatal breastfeeding intention ranged from 92–98% with no significant variation across body mass index (BMI) categories (<25, 25–29.9, ≥30 kg/m^2^) or by parity^9^. According to the Swedish Food Agency’s recommendations from 2011, parents may introduce minimal amounts of solid foods, referred to as ‘tiny tastes’, from four months of age^10^. However, a Swedish study has shown that nearly half of all infants were introduced to tiny tastes at 3–4 months of age, and that the introduction of tiny tastes had an association with shortened breastfeeding duration^11^.

As stated by the WHO, the term postnatal encompasses all issues concerning the woman and newborn after birth^12^. The early postnatal period refers to the first week following birth^13^. The midwife attending birth performs a comprehensive assessment of physical, social, and psychological health and risk factors. Based on the assessment, an individualized care plan for the first week is designed. Depending on the need for care, in-hospital or home-based postnatal midwifery care (HBPMC) is offered. For women and newborns identified with obstetrical or neonatal risk factors, this plan also includes an evaluation by an obstetrician or pediatrician. Subsequently, the family is referred to the appropriate level of care in accordance with the established care plan. The average postnatal length of stay in hospital is approximately two days following an uncomplicated vaginal birth and three days after a cesarean section^14^. One week after birth, the infant is enrolled in the national child healthcare program with standardized follow-up of health and development^15^.

The number of hospitals offering HBPMC in Sweden has increased in recent years. Johansson et al.^16^ found that women in Sweden who received HBPMC reported high levels of satisfaction, and most indicated HBPMC as their preferred model of postnatal care for future births. Home-based postnatal midwifery care in Sweden and Norway has been evaluated by parents as meeting individual needs, with midwives perceived as highly available and present^17,18^. The home environment has been described as comfortable and secure for building infant bonding and supporting partner involvement^17,19^.

Several studies have shown that HBPMC is a model of care that is appreciated by women and their partners^16-19^. However, research investigating HBPMC and its association with exclusive breastfeeding is lacking. Given that EB is linked to multiple health benefits for both mother and infant^2,3^, and that current EB rates in Sweden remain low, it is of importance to investigate breastfeeding rates among women receiving HBPMC. Moreover, there is a need to identify factors associated with cessation of EB within the first two months after birth. Therefore, the aim of this study was to investigate breastfeeding outcomes at two months after birth in women and newborns receiving HBPMC. Furthermore, to identify risk factors and to describe women’s reported reasons associated with EB cessation within two months after birth.

## METHODS

### Study design

This is a prospective cohort study with an observational design. Data were collected between November 2023 and March 2024 from medical records and by telephone interviews at two months after birth.

### Setting

The study was conducted at two hospitals (Site A and B) in southern Sweden with approximately 4800 (Site A) and 2900 (Site B) annual births^20^, respectively, where the hospitals are providing postnatal care during the first week after birth. Postnatal in-hospital care was available at two levels: a family-centered postnatal care unit for healthy women and newborns, where a partner or close relative may stay, and a specialized postnatal care unit for women and/or newborns requiring additional medical care.

A postnatal midwifery care program, including in-home care, was introduced at Site A in February 2019 and at Site B in October 2023. Together, in 2023, both sites provided in-home postnatal midwifery care to approximately 23% of the families each month. Home-based postnatal midwifery care is available for low-risk mothers with healthy newborns after a minimum of six hours of postpartum in-hospital stay. For women or newborns who required an initial in-hospital stay, transfer to HBPMC could be arranged when risk factors were no longer present.

The home-based postnatal midwifery care program offers the same screening program for newborns and women as in hospital care. For newborns, it includes screening for hyperbilirubinemia, the phenylketonuria (PKU) test, the Otoacoustic Emissions (OAE) test, breastfeeding support, and scheduling formula feeding when needed, weight, and newborn head-to-toe physical examination. For women, it involves vaginal tear examination, assessment of recovery, physical changes after birth (micturition and feces, uterus involution and postpartum bleeding), and emotional health, including birth experience. The HBPMC model of care is organized with daily contact by telephone with assessment of overall health for both the newborn and mother, and in-home care when required during the first postnatal week, meeting the unique needs of each family. The in-home care is focused on breastfeeding observation and support, including guidance and testing different breastfeeding positions, observing latching and breastfeeding behavior.

### Participants

**Figure 1:**
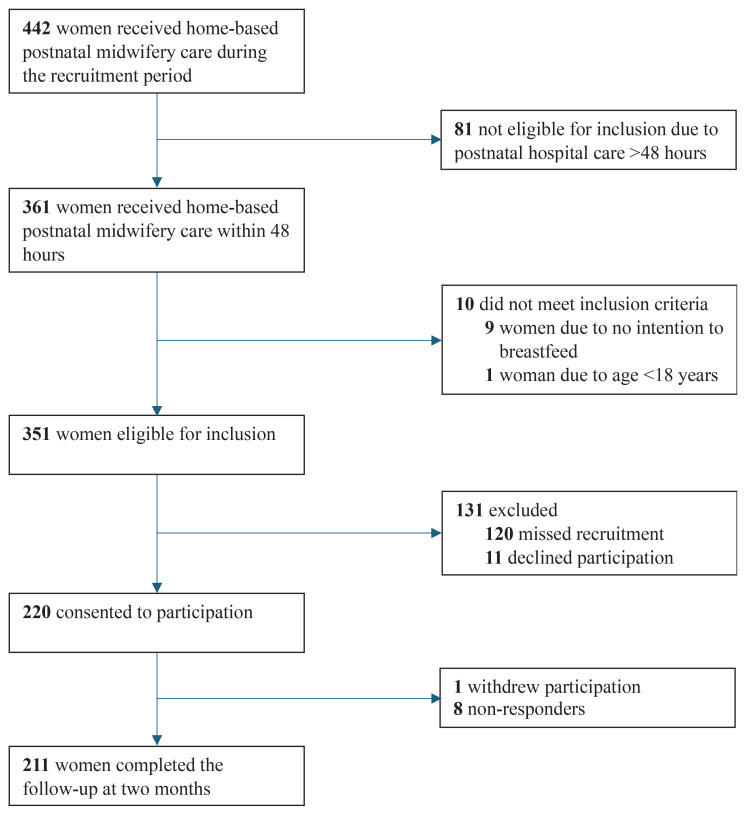
Flow chart of women participating in the study

Inclusion criteria were being at least 18 years of age, having a prenatal intention to breastfeed, and being referred to HBPMC within 48 hours after birth (Figure 1). Women were recruited consecutively and were invited to participate in the study at the last visit before referral to the national child healthcare program. However, due to staff constraints, not all women eligible were approached. Oral and written information about the study was provided by the caregiving midwife. For participants who did not read Swedish, information was given orally by a language interpreter. Written consent was obtained from all participants, and they were informed of the possibility of withdrawing at any time.

### Data collection

Data retrieved from medical records were: maternal age, parity (primiparity, multiparity), country of birth, a documented need for an interpreter (yes, no), prenatal breastfeeding intention (yes, no), mode of birth (spontaneous vaginal birth, instrumental vaginal birth or cesarean section), postpartum hemorrhage >1000 mL (yes, no), severe perineal trauma (yes, no), level of postnatal care (in-hospital, HBPMC), number of in-home visits (1, 2 or ≥3), neonatal birth weight, neonatal weight loss (%) during the first week and, if relevant, indication for formula supplementation. Data on nipple wounds/cracked nipple, delayed onset of lactation, and breastfeeding during the first week after birth were summarized and documented by the midwife at the last visit in the HBPMC program. Follow-up telephone interviews were conducted by the first author two to three months after birth to determine breastfeeding outcome at two months. During the telephone interviews, the following questions were posed: ‘What did you feed your baby at two months of age?’. The answers were categorized into exclusive breastfeeding, partial breastfeeding, and no breastfeeding. In cases of ceased EB, questions about when and the reasons for EB cessation were posed: ‘If you do not exclusively breastfeed, when did you first introduce formula?’ and ‘Many reasons could be given for the introduction of formula, would you like to tell me about your reason?’.

The women who did not speak Swedish were given the possibility of having an interpreter during the telephone interview. However, all included women preferred a family member to act as interpreter. The women received a text message in advance by telephone with information about the upcoming follow-up and interview. Women who did not respond to the initial telephone call were contacted again on up to two additional occasions. Those who did not respond to any of the telephone calls received an e-mail including the interview questions and were given the option to respond by e-mail. Two reminders were sent by email to non-responders.

### Outcome variable

Breastfeeding outcome at two months after birth was investigated and dichotomized into ‘exclusively breastfeeding’ and ‘not exclusively breastfeeding’ (which includes partial breastfeeding and no breastfeeding). Exclusive breastfeeding was defined according to the WHO definition, i.e. the newborn receiving only breastmilk and no other food or liquid, except for vitamins, minerals, and oral medications, in the past seven days. Partial breastfeeding at two months was defined as breastfeeding while also providing one or more formula feedings within the past seven days, which is the definition used when reporting national breastfeeding statistics^21^.

### Explanatory variables

To identify associations with breastfeeding outcome at two months after birth, the following variables were investigated: age, parity, BMI, country of birth, a documented need for an interpreter, breastfeeding status at discharge from HBPMC, observed nipple wounds/cracked nipples, and delayed onset of lactation. Onset of lactation was considered delayed if the woman reported that her breasts had not become noticeably engorged within 72 hours after birth. Breastfeeding status at discharge was categorized as exclusive breastfeeding (including expressed breast milk) or partial breastfeeding. Partial breastfeeding was defined as any supplemental feeding given during the first week after birth, either with prior or ongoing formula supplementation at discharge from HBPMC. The variables were dichotomized as follows; age (<30, ≥30 years), parity (primiparous, multiparous), pre-pregnancy BMI (<30, ≥30 kg/m^2^), country of birth (Sweden, other), a documented need for an interpreter (yes, no), nipple wounds/cracked nipples (yes, no), delayed onset of lactation (yes, no) and breastfeeding at discharge from HBPMC (EB, partial breastfeeding).

### Data analysis

Reasons given for EB cessation were provided in response to the open-ended question: ‘What did you feed your baby at two months of age?’. The reasons for EB cessation were then identified as: perceived insufficient milk supply, breastfeeding challenges, medical indications, perceived breastfeeding barriers, or other reasons. The categories were inspired by Reynolds et al.^22^ and covered the examples provided by women. Multiple reasons could be reported.

Descriptive statistics were used to present the study sample. To identify factors associated with EB cessation within two months, univariable logistic regression was performed to calculate crude odds ratios with a 95% confidence interval (CI). Thereafter, variables with statistical significance were analyzed in a multivariable logistic regression model to identify factors independently associated with EB cessation before two months, using adjusted odds ratios (AORs) with 95% confidence intervals (CIs). The analyses were conducted using IBM SPSS software (version 29).

## RESULTS

Out of the 442 women who received home-based postnatal midwifery care during the recruitment period, 351 women were eligible for inclusion, and 220 (62.7%) consented to participate. One woman withdrew, leaving 219 women in the study. In total, 211 of the 219 women responded (96.3%), of which 196 participated in telephone interviews, and 15 provided their responses via e-mail. In total, 60% (n=132) of the women were included from Site A, while 40% (n=87) were included from Site B. Approximately one in four women was born outside of Sweden, representing a total of 33 different countries. Prenatal intention to breastfeed exclusively was documented for 97.3% of the women, while 2.3% reported the intention to partially breastfeed (Table 1). Most women had a spontaneous vaginal birth (94.5%).

**Table 1:** Background characteristics of participants receiving HBPMC, prospective cohort study, Sweden, 2023–2024 (N=219)

*Characteristics*	*n (%)*
**Maternal age** (years)	
Mean ± SD	32.3 ± 4.5
<30	54 (24.7)
≥30	165 (75.3)
**Parity**	
Primiparous	55 (25.1)
Multiparous	164 (74.9)
**Pre-pregnancy BMI** (kg/m^2^)	
Mean ± SD	24.8 ± 4.4
<30	185 (84.5)
≥30	31 (14.2)
Missing data	3 (1.4)
**Country of birth**	
Sweden	149 (68)
Other*	59 (27)
Missing data	11 (5.0)
**Documented need for interpreter**	
Yes	14 (6.4)
No	205 (93.6)
**Intention to breastfeed**	
Exclusively	213 (97.3)
Partially	5 (2.3)
Missing	1 (0.5)

HBPMC: home-based postnatal midwifery care. BMI: body mass index.

* African n=1, Asian n=7, European n=27, Middle Eastern n=23, South American n=1.

At referral to child healthcare when discharged from HBPMC, 5% of the newborns had ongoing formula supplementation, and 9% had received formula supplementation during the first week after birth (Table 2). Two months after birth, 83.9% were exclusively breastfeeding, 11.8% were partially breastfeeding, and 4.3% had ceased to breastfeed. Exclusive breastfeeding cessation before two months was significantly associated with introduction of formula during the first week after birth (OR=5.83; 95% CI: 2.48–13.68), having a BMI ≥30 (OR=2.77; 95% CI: 1.14–6.77), and country of birth other than Sweden (OR=2.57; 95% CI: 1.17–5.67) (Table 3). These associations remained when analyzed in a multivariable logistic regression model: introduction of formula during the first week after birth (AOR=5.89; 95% CI: 2.31–15.02), BMI ≥30 (AOR=3.55; 95% CI: 1.27–9.94), and country of birth other than Sweden (AOR=2.39; 95% CI: 1.01–5.67) (Table 4).

**Table 2:** Labor, birth and breastfeeding data of participants receiving HBPMC, prospective cohort study, Sweden, 2023–2024 (N=219)

*Characteristics*	*n (%)*
**Mode of birth**	
Spontaneous vaginal birth	207 (94.5)
Instrumental vaginal birth	10 (4.6)
Cesarean section	2 (0.9)
**Postpartum hemorrhage** (>1000 mL)	
Yes	10 (4.6)
No	209 (95.4)
**Severe perineal trauma**	
Yes	2 (0.9)
No	217 (99.1)
**Highest level of in-hospital postnatal care before referral to in-home care**	
Birth unit	51 (23.3)
Family-centered postnatal care unit	74 (33.8)
Specialized postnatal care unit	93 (42.5)
None in hospital (home birth)	1 (0.5)
**Number of home visits**	
1	55 (25.1)
2	101 (46.1)
≥3	63 (28.8)
**Nipple wounds/cracked nipple**	
Yes	52 (23.7)
No	167 (76.3)
**Delayed onset of lactation**	
Yes	26 (11.9)
No	193 (88.1)
**Breastfeeding at discharge from HBPMC**	
EB (including expressed breast milk)	188 (85.9)
Partial breastfeeding	31 (14.1)
- Breastfeeding with prior formula supplementation	20 (9.1)
- Breastfeeding with ongoing formula supplementation	11 (5.0)

EB: exclusive breastfeeding. HBPMC: home-based postnatal midwifery care.

**Table 3:** Univariable logistic regression analyses of factors associated with EB cessation before two months after birth in women receiving HBPMC, prospective cohort study, Sweden, 2023–2024 (N=211)

*Variables*	*Exclusive breastfeeding n (%)*	*Exclusive breastfeeding cessation n (%)*	*OR (95% CI)*
**Age** (years)			
<30 (ref.)	39 (81.3)	9 (18.6)	1.0
≥30	138 (84.7)	25 (15.3)	0.79 (0.34–1.82)
**Parity**			
Primiparous	42 (80.8)	10 (19.2)	1.33 (0.59–3.03)
Multiparous (ref.)	135 (84.9)	24 (15.1)	1.0
**BMI** (kg/m^2^) (N=208)			
<30 (ref.)	154 (86.0)	25 (14.0)	1.0
≥30	20 (69.0)	9 (31.0)	2.77 (1.14–6.77)
**Country of birth** (N=200)			
Sweden (ref.)	128 (88.3)	17 (11.7)	1.0
Other	41 (74.5)	14 (25.5)	2.57 (1.17–5.67)
**Documented need for interpreter**			
No	166 (83.8)	32 (16.2)	NA
Yes	11 (84.6)	2 (15.4)	
**Introduction of formula during the first week after birth**			
No (ref.)	160 (88.4)	21 (11.6)	1.0
Yes	17 (56.7)	13 (43.3)	5.83 (2.48–13.68)
**Nipple wounds/cracked nipple**			
No (ref.)	134 (83.8)	26 (16.3)	1.0
Yes	43 (84.3)	8 (15.7)	0.96 (0.40–2.27)
**Delayed onset of lactation**			
No (ref.)	158 (84.9)	28 (15.1)	1.0
Yes	19 (76.0)	6 (24.0)	1.78 (0.65–4.85)

EB: exclusive breastfeeding. HBPMC: home-based postnatal midwifery care. BMI: body mass index. OR: crude odds ratio. NA: not applicable.

**Table 4:** Multivariable logistic regression analyses of factors associated with cessation of EB before two months, participants receiving HBPMC, prospective cohort study, Sweden, 2023–2024 (N=197)

*Factors for EB cessation before two months*	*AOR (95% CI)*
**Introduction of formula during the first week after birth**	
No (ref.)	1.0
Yes	5.89 (2.31–15.02)
**BMI** (kg/m^2^)	
<30 (ref.)	1.0
≥30	3.55 (1.27–9.94)
**Country of birth**	
Sweden (ref.)	1.0
Other	2.39 (1.01–5.67)

EB: exclusive breastfeeding. HBPMC: home-based postnatal midwifery care. BMI: body mass index. AOR: adjusted odds ratio.

Indications reported for formula supplementation during the first week after birth included gestational diabetes, hypoglycemia, low Apgar score, respiratory distress, large for gestational age, uncertainty regarding the newborn’s urinary output, the mother’s request due to a fussy newborn, the mother’s intention to partially breastfeed, or supplementation given without a documented indication. Weight loss was not an indication for formula supplementation, as none of the newborns lost more than 10% of their birth weight during the first week after birth; mean neonatal weight loss: 2.2% (SD=3.5).

**Figure 2:**
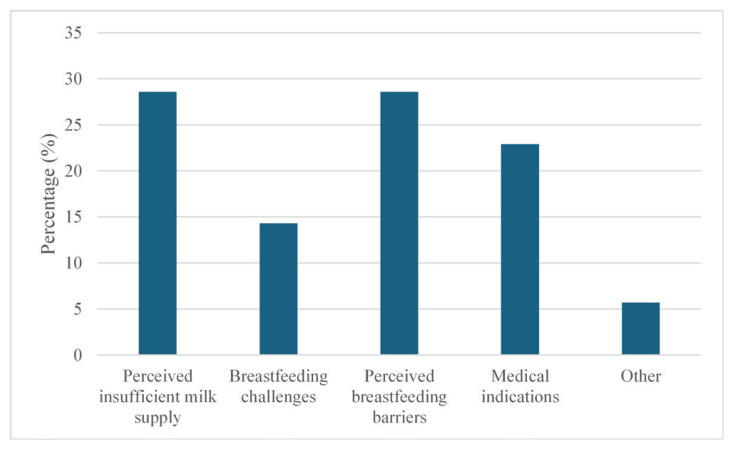
Women’s reported reasons for EB cessation before two months

Women’s reported reasons for EB cessation before two months were classified into five categories (Figure 2):

- Perceived insufficient milk supply – reported by 28.6% of the women, included a fussy infant, breastfeeding too often, and not having enough milk.
- Breastfeeding challenges – were stated by 14.3% and included the infant’s lack of interest or poor latch, difficulties using a nipple shield, and the infant not breastfeeding sufficiently due to illness.
- Perceived breastfeeding barriers – reported by 28.6%, include having other children to care for, a lack of time to breastfeed, a preference for sharing feeding responsibilities between the parents, a desire for improved sleep at night, previous negative experiences with breastfeeding, the infant being calmer in the evenings with formula feeding, and a need for more personal space.
- Medical indications – reported reasons (22.9%) included insufficient infant weight gain, infant weight loss, recommendations from healthcare staff, maternal infection, and infant allergies.
- Other – reported reasons (5.7%) included separation or loss of a close relative affecting the milk supply.

## DISCUSSION

The rate of exclusive breastfeeding at one week after birth remained largely stable, with only a slight decrease up until two months after birth. Factors significantly associated with EB cessation were the introduction of formula during the first week after birth, having a BMI ≥30, or being born outside of Sweden. The women’s most frequently reported reasons for EB cessation were perceived breastfeeding barriers or a perceived insufficient milk supply.

Exclusive breastfeeding during the first week after birth was associated with a higher likelihood of maintained exclusive breastfeeding at two months after birth, compared with newborns who were introduced to formula during the first postnatal week. Breastfeeding guidance and support during the early days following birth, before breastfeeding is established, have been identified as crucial to support breastfeeding^23^. Concerns about breastfeeding, particularly infant feeding difficulties and perceived low milk quantity at days 3 and 7 after birth, have been associated with breastfeeding cessation within two months after birth^24^. By offering HBPMC, breastfeeding support is provided throughout the first postnatal week, in contrast to standard in-hospital care, where mothers are discharged within two to three days. This extended in-home support allows for identification and support of women experiencing early breastfeeding difficulties, and the maintained high prevalence of EB observed in this study suggests a favorable outcome of HBPMC.

The strongest association with EB cessation within two months after birth was observed for the introduction of formula during the first week after birth. This finding aligns with an American study of primiparous women, where inhospital formula supplementation increased the risk of partial breastfeeding at 30–60 days and cessation by day 60 ^25^. In the present study, EB rates were 86% at referral to child healthcare when discharged from HBPMC. This contrasts with data from the Swedish Pregnancy Register showing that 65% of term, normal-weight infants born to non-diabetic women were exclusively breastfeeding during their hospital stay at site A, and 64% at site B in 2023 ^26^. Furthermore, 84% were breastfeeding exclusively at two months after birth, compared to 59% of women in the catchment area in 2021, according to the National Board of Health and Welfare^8^. Even though indications for formula supplementation during the first week in the present study included medical indications such as maternal gestational diabetes, newborn hypoglycemia, and respiratory distress, the rate of EB may not be entirely comparable with national data. For instance, only women with a prenatal intention to breastfeed were eligible for inclusion in this study. Moreover, women admitted to HBPMC are likely to have a higher prevalence of uncomplicated births and fewer risk factors associated with early breastfeeding cessation.

The rate of pregnant women with a BMI in the obese range is increasing in Sweden^20^. In the present study, women with a BMI ≥30 were more likely to cease exclusive breastfeeding within two months after birth. The findings of a shorter breastfeeding period among overweight and obese women are confirmed in other studies from Australia^22^, Sweden^9^, in an international multi-center study^27^, as well as in a systematic review by Mangrio et al.^28^ on factors affecting breastfeeding cessation before 6 months. Keyes et al.^27^ identified dietary and systemic inflammation as factors mediating an association between overweight and obesity and shortened breastfeeding duration. Obesity has also been shown to be a risk factor for delayed lactogenesis II, i.e. a delay in the onset of milk production beyond 72 hours after birth^29^. A systematic review from 2020, based on research from high-income countries, found further physiological barriers for women who were overweight or obese, such as difficulties with the baby’s latch and positioning and nipple trauma^30^. Furthermore, psychological barriers to EB have been identified among women who were overweight or obese, including perceived insufficient milk supply as well as lower self-confidence in the ability to breastfeed, negative body image, and discomfort of breastfeeding in public^30^. Women with obesity have also been shown to receive less support and information regarding breastfeeding from healthcare professionals^30^. Moreover, a Swedish study on breastfeeding intention and outcome found that multiparous women who were overweight or obese more often expected breastfeeding to be partial, and that fewer women with obesity breastfed exclusively two weeks after birth^9^.

The interplay of physiological and psychological barriers, as well as less support from healthcare professionals, poses significant challenges to EB in women who are overweight or obese. The findings of this study address a need for targeted and extended efforts in mitigating the breastfeeding barriers faced by these women. Providing individualized breastfeeding support may contribute to improved health outcomes for both women and their children.

In the present study, women born outside Sweden originated from 33 countries and represented a heterogeneous group. Few of the women had a documented need for an interpreter, even though the use of professional interpreters in healthcare has improved satisfaction and better communication^31^. A systematic review comparing immigrant and non-immigrant women’s experiences of maternity care showed that immigrant women were less positive about the care they received during the antenatal to the postnatal period^32^. Problems described included language barriers, not enough involvement in decision-making, lack of knowledge about the care organization, and negative attitudes and discrimination from healthcare professionals^32^. Migrant women in Sweden have expressed a greater need for professional interpretation during antenatal and intrapartum care^33^. Increased use of professional interpreters may improve women’s ability to access and understand information provided during breastfeeding support.

Perceived breastfeeding barriers and perceived insufficient milk supply emerged as the most common reasons for EB cessation. Within the category of perceived barriers, women reported motives such as the desire to share feeding responsibilities with others, having more individual time, and allowing time for recovery. Consistent with these findings, previous research has identified perceived insufficient milk supply as one of the leading causes for breastfeeding cessation^22,34-36^. The 2023 Breastfeeding Series synthesizing reviews and case-studies, further reports that self-reported insufficient milk accounts for nearly half of all cases worldwide where women introduce formula supplementation^36^. Self-reported insufficient milk does not distinguish between perceived and actual insufficient milk supply but refers to the woman’s perception of not having enough breastmilk to satisfy the infant’s hunger or support adequate weight gain. Misinterpretation of normal infant behavior as signs of inadequate milk intake from both parents and health professionals addresses concerns and the need for improved education about infants’ developmental behaviour^36^. Infant satiety cues, sucking pattern, and engorgement of breasts have been described as unreliable indicators of milk supply^34^. The perception of insufficient milk supply highlights the importance of providing information already during pregnancy, to strengthen women’s understanding of variations in infant breastfeeding behaviors and patterns, as well as methods for assessing adequate milk intake.

### Strengths and limitations

Strengths of this study include the prospective design and a high response rate of 96% when using follow-up by telephone interviews with an interview guide. Furthermore, as study participation is commonly restricted to individuals proficient in the official language, the inclusion of women not proficient in Swedish or English reflects the multi-ethnic composition of women at site A and increases the generalizability of the findings.

The limitations of this study include the observational design with the risk of confounding, the small study sample, and the regional context, which limits the generalizability of the findings. The higher prevalence of uncomplicated births in the study sample, compared with the general population of birthing women, represents a further limitation. Women who experience uncomplicated births are likely to face fewer breastfeeding-related challenges than those who experience birth by cesarean section^28^. Consequently, the associations observed in this study may not fully capture outcomes following complicated births. The set time frame of the study permitted recruitment for three months. A larger sample size could have allowed for stratified analyses within subgroups, including adjustment for residual confounding. Another limitation of this study is the reliance on self-reported data, which may introduce information bias and misclassification. Moreover, not all women receiving HBPMC during the study period were invited to participate, which may have introduced a selection bias. Furthermore, the use of a family member for interpretation may introduce bias due to miscommunication.

## CONCLUSIONS

A high continuation of exclusive breastfeeding was reported at two months after receiving a home-based postnatal midwifery model of care, suggesting that in-home breastfeeding support during the first week after birth may play an important role. Future interventions providing in-home breastfeeding support, particularly targeting women with identified risk factors, may further improve breastfeeding duration. As perceived insufficient milk supply was one of the most commonly reported reasons for exclusive breastfeeding cessation, providing women with information about normal variations in infant breastfeeding behavior and how to assess adequate milk intake, may be important to support continued exclusive breastfeeding.

Implementing home-based postnatal midwifery care during the first week after birth may enhance exclusive breastfeeding rates and should be considered as part of standard postnatal care. Special attention with targeted support is suggested for women with obesity, women who are foreign-born, and for those whose infants received formula supplementation during the first week after birth, according to associations with cessation of exclusive breastfeeding within two months after birth.

## Data Availability

The data supporting this research are available from the authors on reasonable request.
